# Dual-approach analysis of gut microbiome in patients with type 1 diabetes and diabetic kidney disease

**DOI:** 10.1080/07853890.2025.2531254

**Published:** 2025-07-26

**Authors:** Laura Bunka, Maija Rozenberga, Ivars Silamiķelis, Rihards Saksis, Līga Birzniece, Kaspars Megnis, Aleksejs Fedulovs, Leonora Pahirko, Poļina Zaļizko, Eduards Krustiņš, Jānis Kloviņš, Jeļizaveta Sokolovska, Ilze Elbere

**Affiliations:** a Latvian Biomedical Research and Study Centre, Riga, Latvia; b Faculty of Medicine and Life Sciences, University of Latvia, Riga, Latvia; c Faculty of Science and Technology, University of Latvia, Riga, Latvia; d Gastroenterology, Hepatology and Nutrition Center, Pauls Stradins Clinical University Hospital, Riga, Latvia; e Department of Internal Medicine, Riga Stradins University, Riga, Latvia

**Keywords:** Type 1 diabetes, diabetic kidney disease, 16S rRNA gene analysis, metagenome analysis, continuous glucose monitoring

## Abstract

**Background:**

Type 1 diabetes (T1D) is a multifactorial autoimmune disease mediated by genetic, epigenetic, and environmental factors. Diabetic kidney disease (DKD) is a major complication of diabetes mellitus which affects 30–40% of T1D patients. Increasing evidence suggests the significant role of the microbiome in the progression of both T1D and DKD.

**Materials and methods:**

Here we recruited 76 T1D patients and 22 healthy controls and combined data from sigmoid colon biopsy samples analysed with V3–V4 region amplification of 16S rRNA gene and shotgun metagenomics data obtained from faecal samples. Additionally, we compared T1D patients with and without progression of DKD.

**Results:**

We observed significant differences within both sample types at various taxonomic and functional levels. T1D patient microbiota detected using biopsy samples had a lower abundance of the *Bacteroides* genus when compared to healthy controls. Significantly, despite only a few taxonomic differences patients with and without DKD progression were vastly different at the functional pathway level within the faecal samples - we observed 2 and 61 enriched pathways in these groups. respectively, with several of these pathways linked to the mediation of renal function.

**Conclusion:**

Altogether, we present novel data about microbial signatures relevant to T1D and DKD progression, which partly supports previous data and also presents possible tissue type or population-specific elements. DKD progression is characterized with significant differences within the functional level of the gut microbiome.

## Introduction

The gut microbiome represents the most diverse and densely populated microbial community per unit volume in the human body. It plays a critical role in health and disease, contributing to various essential physiological functions. Many of these microorganisms are bacteria residing in the colon [1]. Studies have shown that an altered gut microbiome is associated with the development of various diseases [2,3].

Diabetes is a chronic metabolic disease characterized by elevated blood glucose levels, which, if left uncontrolled, can lead to severe health complications, including acute issues such as diabetic ketoacidosis, which can be life-threatening, as well as, over time, a range of cardiovascular diseases, chronic kidney disease, nerve damage and blindness [4–6]. Diabetes has become one of the most serious and widespread health problems globally and its prevalence continues to increase. In 2021, the global prevalence of diabetes among people aged 20–79 exceeded 500 million [7].

Type 1 diabetes (T1D), an autoimmune disease characterized by the body’s inability to produce insulin, affects 5–10% of all diabetes patients [8]. This condition results from an interplay between genetic and environmental factors. In recent years, the role of gut microbiome in disease development, particularly its taxonomic and functional profile changes, has been the most extensively studied among environmental factors; however, many uncertainties remain. While diabetes is currently incurable, timely diagnosis and appropriate treatment can improve the quality of life, reduce the risk of complications and slow the progression of the disease [4,9]. A deeper understanding of how the gut microbiome and other influencing factors contribute to the development of type 1 diabetes and its complications is crucial for improving disease prediction and therapeutic applications [3,10].

The most used methods for studying the gut microbiome are amplicon and metagenomic sequencing [11,12]. To obtain a more comprehensive understanding of the human gut microbiome, it is recommended to combine multiple analytical methods. Such studies are relatively rare due to higher costs and complexity, but in recent years, the combination of these methods has become increasingly popular for fully understanding microbial communities and their roles in various environments or diseases [13,14]. Additionally, there are two main types of samples commonly analysed in gut microbiome studies: faecal samples and intestinal biopsies. While faecal samples provide insights into the luminal microbiota, biopsies allow for the investigation of the mucosal-associated microbiota, offering a more localized perspective of microbial communities directly interacting with the intestinal epithelium [15].

Therefore, considering previous studies, we aimed to use both microbiome analysis methods to conduct an in-depth investigation of the taxonomic and functional changes in the gut microbiome of patients with type 1 diabetes, compared to healthy individuals, while also examining the impact of glucose levels and different stages of severity of diabetic kidney disease (DKD). By employing these analysis methods to study both faecal and biopsy samples, this study provides deeper insights into the role of the gut microbiome in T1D and DKD and present novel data about microbial signatures related to these diseases in a cohort from the Latvian population.

## Patients, materials and methods

### Study design

The present study is part of the longitudinal LatDiane study, which commenced in 2013 and is a participant in the international InterDiane consortium. LatDiane focuses on recruiting adult patients with T1D who were diagnosed before the age of 40, started insulin treatment within one year of diagnosis and have C-peptide levels <0.3 nmol/l. Patients with a history of chronic kidney disease apart from DKD or documented treatment with oral antihyperglycemic treatment are excluded from LatDiane [16,17]. Follow-up visits occur every three years. The study protocol for the overall LatDiane study and the sub-study reported herein have received approvals from the Latvian Central Ethics Committee (Riga, Latvia) under permission No. 01-29.1/3 (dated, 10 July 2013), Nr.A-17/19-10-17 (dated, 17 October 2019), Nr. 01-29.1.2/3274 (dated 23 April 2021). All participants provided written informed consent and were enrolled in the Genome Database of the Latvian Population (LGDB) with recruitment organized according to previously established practices [18].

The study included patients with type 1 diabetes mellitus (T1D) with stable or progressive diabetic kidney disease (DKD) and healthy volunteers without diabetes and other chronic diseases. Recruitment for this study took place between 4 November 2021 and 20 February 2023. All T1D patients were using insulin for the treatment of hyperglycaemia. Control subjects were generally healthy spouses and friends of enrolled individuals with type 1 diabetes, university personnel and other volunteers. Information about the study was disseminated *via* the University of Latvia’s webpage and social media. The age and gender distribution of the control group was matched that of the diabetes patients. Progressive DKD was defined as a decline in estimated glomerular filtration rate (eGFR) of ≥ −3 mL/min/year, based on at least three serum creatinine measurements from visits between 2013 and 2016 and/or progression in albuminuria status (e.g. from normoalbuminuria to microalbuminuria or macroalbuminuria, or to end-stage renal disease). Stable or non-progressive DKD was defined as an eGFR decline of no more than −3 mL/min/year and no progression in albuminuria.

For continuous glucose monitoring (CGM), FreeStyle Libre Pro iQ Sensors (Abbott GmbH) were utilized as diagnostic, ‘blinded’ glucose sensors. Each participant wore the sensor continuously for a period of 14 days. We analysed data on average glucose levels, glucose variability (CV – coefficient of variation), estimated glycated haemoglobin (A1c), glucose management indicator (GMI) and the percentage of time spent above the target range (%TAR), within the target range (%TIR) and below target range (%TBT), low glucose events and average duration of low glucose events. Based on current guidelines for individuals with T1D, %TIR was defined as glucose levels between 3.9 and 10 mmol/L [19].

Exclusion criteria for all participants included a history of chronic kidney disease unrelated to DKD, inflammatory bowel disease (IBD), such as Crohn’s disease or ulcerative colitis, coeliac disease, or any acute intestinal infection within the last two months. Participants were recruited in collaboration with gastroenterologists and endocrinologists at Pauls Stradiņš Clinical University Hospital (PSKUS).

### Sample and data collection

Within the study, patients and controls completed a gastrointestinal symptom questionnaire and underwent relevant analyses, after which certified medical personnel selected candidates for colonoscopy. Indications for colonoscopy included the following criteria: unexplained weight loss; clinical suspicion of inflammatory bowel disease; unexplained abdominal pain; unexplained anaemia (especially iron-deficiency anaemia); unexplained changes in bowel habits (such as diarrhoea, constipation, or variable bowel movements); the presence of mucus in the faeces; family history of inflammatory bowel disease or colorectal cancer; personal history of inflammatory bowel disease or polyps; elevated faecal calprotectin levels (> 50 µg/g); or screening colonoscopy for individuals over 50 years of age. Prior to the collection of faecal samples, all participants provided data on various anthropometric parameters, dietary habits, medical conditions, medications, test results and other relevant indicators. Prior to colonoscopy, they were appropriately prepared, using the laxative ‘Moviprep’ to cleanse the bowel. The preparation process for T1D patients included enhanced glycaemic control and adjusted insulin dosing to account for the reduced food intake five to seven days before the procedure, minimizing the risk of hypoglycaemia.

Certified medical personnel collected intestinal biopsy samples during fibrocolonoscopy by inserting the endoscope approximately 40–50 cm deep to obtain samples from the sigmoid colon (approximately midsection). Biopsy samples were collected from 27 individuals: 7 healthy controls and 20 T1D patients, 4 of whom had progressive DKD. Following collection, the biopsy samples were immediately frozen at −20 °C and transported to the research laboratory within 24 h, where they were stored at −80 °C until further processing and analysis. For each of the 27 individuals, one to three samples that passed quality control were sequenced, resulting in a total of 71 16S rRNA gene V3–V4 region libraries. During the bioinformatic processing of sequencing data, replicates of each individual’s intestinal biopsy samples were combined.

Faecal samples were collected from 92 individuals: 18 healthy individuals and 74 T1D patients, 27 of whom had progressive DKD. Faecal samples were collected at home in a sterile tube without a buffer. Participants were instructed to deliver the samples to the research or clinical laboratory within 24 h, where the samples were initially stored at −20 °C. After delivery to the research laboratory, the samples were stored at −80 °C until further processing and preparation of metagenome libraries.

Taking into account the previously described indications for performing a colonoscopy, both faecal and intestinal biopsy samples were obtained from 20 individuals: 3 healthy individuals and 17 patients with T1D, 4 of whom had progressive DKD. Faecal samples from these 20 participants were collected 7 ± 3 months prior to the collection of intestinal biopsy samples.

### Microbial DNA processing of intestinal biopsy samples

Microbial DNA from intestinal biopsy samples was extracted under sterile conditions in a Biosafety Level 2 cabinet using FastDNA Spin Kit for Soil (MP Biomedicals, Santa Ana, CA, USA) and FastPrep Instrument following the instructions of the manufacturer. DNA concentrations were evaluated using a Qubit 2.0 fluorometer (Thermo Fisher Scientific, Waltham, MA, USA). The V3–V4 hypervariable region of the bacterial 16S rRNA gene was PCR-amplified using Phusion U Multiplex PCR Master Mix (Thermo Scientific, USA) and a 341 F/805R primer pair (Supplementary Table 1). Embedded sample identification sequences were added during a second PCR using appropriate oligonucleotides (Supplementary Table 2). Several blank controls were included at each step. Successful amplification of samples and purity of blank controls were determined by agarose gel electrophoresis. The PCR amplicons were purified using NucleoMag magnetic beads (Macherey-Nagel, Düren, Germany) and their quantity and quality were evaluated with the Agilent High Sensitivity DNA kit and Bioanalyzer 2100 instrument (Agilent Technologies, Santa Clara, CA, USA). Sample sequencing was performed on the Illumina MiSeq platform using the MiSeq Reagent Kit v2 (500-cycles) (Illumina, USA), obtaining at least 100 thousand sequencing reads per sample. All samples from each study participant were placed on the same chip to prevent a batch effect.

### Microbial DNA processing of faecal samples

Microbial DNA from the faecal samples was extracted using the automated platform MGISP-960 (MGI Tech) and the MagPure Stool DNA LQ Kit (MGITech) reagent kit. Further shotgun metagenomic library preparation was done by fragmenting the DNA at 400 bp (Covaris), clean-up with MGIEasy DNA Clean Beads and following the manual of the MGIEasy Universal DNA Library Prep Set (MGI Tech Co. Ltd). The library preparation included the following sample processing steps: (1) end repair and A-tailing, (2) Barcode Adapter ligation and clean-up, (3) amplification and clean‑up. Blank controls for DNA extraction, library preparation and sequencing were included in sample analysis batches as part of laboratory standard operating procedures for shotgun metagenomics. After that, the appropriate count of libraries (to obtain the planned read count) was pooled and each pool was normalized to 1 pmol in a volume of 48 μl. The pooled libraries were further annealed and circularized with splint oligo and the final circularization product was cleaned-up. Furthermore, DNA nanoballs were created with rolling circle amplification. The end-products were sequenced using DNBSEQ-G400RS sequencing platform (FCL PE150), obtaining at least 20 M reads per sample.

### Data processing

Quality control of the 16S rRNA amplicon sequencing data was initially performed using FastQC (v0.11.9) and MultiQC (v1.14) [20] software. The data were subsequently imported into the QIIME2 environment (v2022.2) [21, p. 2], where the taxonomic composition (up to genus level) was determined using the SILVA rRNA database (v138.1) [22]. Contaminant identification and filtering were conducted in the RStudio environment (v4.2) using the decontam package (v1.18.0) [23]. Taxa that were relatively more abundant in negative controls compared to the actual samples were identified as contaminants.

For the metagenomic sequencing data, adapter trimming was performed using cutadapt (v1.16), and read trimming was conducted with fastp (v0.20.0) using default settings, retaining reads with a minimum length of 100 base pairs. Reads aligned to the human genome were filtered out with bowtie2 (v2.3.5.1) using the GRCh38 reference genome. Taxonomic classification of microorganisms was performed with kraken2 (v2.0.8) [24, p. 2] using the Unified Human Gastrointestinal Genome (UHGG) catalogue [25], with a confidence score threshold of 0,1. The composition of reads was refined to the genus and species levels and quantified with bracken (v2.7) [26], applying a read count threshold of 10. Functional analysis was conducted using the HUMAnN3 pipeline (v3.8) with the UniRef90 database [27, p. 3].

### Data analysis

Group comparability between T1D patients and healthy controls was assessed by comparing age, gender and BMI. Distributions of age and BMI were assessed using the Shapiro–Wilk test and visualized with Q–Q plots generated using the qqplotr package (v0.0.6) in RStudio (v4.3.2). Based on distribution characteristics, group differences were tested using either Student’s t-test or the Wilcoxon test. Gender differences were assessed using the chi-squared test or Fisher’s exact test, as appropriate.

Six CGM metrics – average glucose levels, glucose variability (CV - coefficient of variation), percentage of time spent above target range (%TAR), within target range (%TIR), below target range (%TBR) and the frequency of low glucose events – were used to cluster T1D patients into subgroups: CGM cluster 1 and CGM cluster 2. The optimal number of clusters was determined to be two, based on the most frequent cluster count among 30 different optimal cluster indices calculated using the NbClust function in R. Hierarchical agglomerative clustering was performed using Ward’s minimum variance linkage method with Euclidean distance. To address issues of high inter-variable correlation, principal component analysis (PCA) was applied prior to clustering.

After bioinformatic processing of both 16S rRNA amplicon sequencing taxonomic data and metagenomic sequencing taxonomic and functional data, one T1D patient was excluded from the metagenomic data analysis due to low sequencing data quality. Taxonomic profile data were converted to proportions and the following statistical analyses were then performed. In the functional data analysis, unmapped and unintegrated functions were not included.

Alpha diversity was assessed by calculating the Shannon diversity index using the vegan package (v2.6.4) in RStudio (v4.3.2) [28]. Comparisons of alpha diversity index values between study groups were conducted using the non-parametric Wilcoxon test, with the results visualized through box plots created using the ggpubr package (v0.6.0).

Beta diversity comparisons between samples were performed using non-metric multidimensional scaling (NMDS) with Bray-Curtis distances, utilizing the vegan (v2.6.4) and ggplot2 (v3.4.4) packages [29]. Permutational multivariate analysis of variance (PERMANOVA) was conducted with 9999 permutations [30] using the vegan (v2.6.4) and tidyverse (v2.0.0) packages in RStudio.

To identify associations of taxonomic composition and functions with study groups and other phenotypic traits, Microbiome Multivariable Associations with Linear Models (MaAsLin) analysis was performed using the MaAsLin2 package (v1.16.0) in RStudio [31]. Graphical representations were generated with the ggplot2 package (v3.4.4). To minimize false-positive results and enhance statistical power, taxa filtering was applied before MaAsLin2 analysis using Command Prompt (v10.0.22631.3593) and Python (v3.12), with taxonomic occurrence thresholds set to require that each identified microorganism or function be present in at least 10% of samples (sample threshold = 0,1) and represent at least 0,01% in each sample where it is identified (abundance threshold = 0.0001). MaAsLin2 analysis was performed, accounting for batch effects by including the grouping of samples based on microbial DNA extraction batches from intestinal biopsy samples and sequencing batches of metagenomic libraries from faecal samples as random effects. PERMANOVA with 9999 permutations was performed using the vegan (v2.6.4) and tidyverse (v2.0.0) packages in RStudio to identify significant phenotypic traits to include in the MaAsLin2 analysis model. The selection of phenotypic traits for this analysis was guided by expert judgement.

The significance threshold for p-values and adjusted p-values in PERMANOVA analysis was set at 0,05, and the q-value threshold for the MaAsLin2 analysis was set at 0,25, according to other microbiome studies and recommendations by the MaAsLin2 developers [31].

To assess the relationship between genera across 17 T1D patients, from which both biopsy and faecal samples were collected, Spearman’s correlation analysis was performed on the filtered taxonomy data at the genus level from biopsy 16S rRNA and faecal metagenomics samples. The results were visualised using a heatmap created with the pheatmap package (v1.0.12) in RStudio.

## Results

### Characterization of the cohort

In total, we recruited 76 T1D patients and 22 healthy controls, from whom 17 T1D patients and 3 healthy controls had both biopsy and faecal samples. Comparisons of gut microbiome taxonomic and functional profiles were performed in the following contrasts: (1) T1D patients vs healthy individuals; (2) T1D patients with progression of DKD vs T1D patients with no progression of DKD; (3) T1D patients in CGM cluster 1 vs T1D patients in CGM cluster 2. More detailed characterization of analysed participants and the specified subgroups is depicted in Table 1 and Table 2.

**Table 1.** Characteristics of intestinal biopsy sample donors.

Characteristic		T1D patients (*n* = 20)				
	Healthy individuals (*n* = 7)		T1D patients with no progression of DKD (*n* = 16)	T1D patients with progression of DKD (*n* = 4)	T1D patients in CGM cluster 1 (*n* = 5)	T1D patients in CGM cluster 2 (*n* = 12)
Women / men, *n* (%)	4 (57%) / 3 (43%)	11 (55%) / 9 (45%)	8 (50%) / 8 (50%)	3 (75%) / 1 (25%)	2 (40%) / 3 (60%)	7 (58%) / 5 (42%)
Age (years), mean ± STDEV	40 ± 6	46 ± 11	45 ± 11	49 ± 13	40 ± 13	47 ± 12
BMI (kg/m^2^), mean ± STDEV	24,88 ± 5,19	26,76 ± 5,29	26,58 ± 5,73	27,48 ± 3,49	24,00 ± 5,10	26,51 ± 3,89
Duration of T1D diagnosis (years), mean ± STDEV	–	30 ± 11	30 ± 12	29 ± 10	18 ± 5	33 ± 11
Antibiotics used in the last two months, *n* (%)	0	3 (15%)	1 (6%)	2 (50%)	0	3 (25%)
Diarrhea in the past week, *n* (%)	1 (14%)	1 (5%)	1 (6%)	0	0	1 (0,08%)

Abbreviations : T1D: type 1 diabetes; DKD: diabetic kidney disease; CGM: continuous glucose monitoring; n: number of individuals; STDEV: standard deviation; BMI: body mass index.

**Table 2.** Characteristics of faecal sample donors.

Characteristic		T1D patients (*n* = 73)				
	Healthy individuals (*n* = 18)		T1D patients with no progression of DKD (*n* = 47)	T1D patients with progression of DKD (*n* = 26)	T1D patients in CGM cluster 1 (*n* = 27)	T1D patients in CGM cluster 2 (*n* = 44)
Women / men, *n* (%)	13 (72%) / 5 (28%)	44 (60%) / 29 (40%)	26 (55%) / 21 (45%)	18 (69%) / 8 (31%)	18 (67%) / 33 (%)	24 (55%) / 20 (45%)
Age (years), mean ± STDEV	38 ± 9	44 ± 12	42 ± 11	49 ± 12	46 ± 13	43 ± 11
BMI (kg/m^2^), mean ± STDEV	23,64 ± 2,75	25,63 ± 4,16	25,73 ± 4,21	25,43 ± 4,15	26,21 ± 4,22	25,08 ± 3,93
Duration of T1D diagnosis (years), mean ± STDEV	–	26 ± 11	24 ± 11	30 ± 12	26 ± 10	26 ± 12
Antibiotics used in the last two months, *n* (%)	0	11 (15%)	8 (17%)	3 (12%)	3 (11%)	8 (18%)
Diarrhea in the past week, *n* (%)	0	10 (14%)	7 (15%)	3 (12%)	3 (11%)	6 (14%)

Abbreviations: T1D: type 1 diabetes; DKD: diabetic kidney disease; CGM: continuous glucose monitoring; *n*: number of individuals; STDEV: standard deviation; BMI: body mass index.

To minimize potential bias due to unequal group sizes, we verified that the T1D patients and healthy control group are matched by such factors as age, gender and BMI. No significant differences were found for gender (biopsy samples: Fisher’s exact test, *p* = 1.000; faecal samples: χ^2^(1) = 0.44, *p* = .505), age (biopsy samples: t(25) = −1.26, *p* = .221; faecal samples: *W* = 465, *p* = .056), or BMI (biopsy samples: t(25) = −0.81, *p* = .426; faecal samples: t(89) = −1.92, *p* = .058). These results indicate sufficient matching of demographic characteristics between the groups.

Furthermore, in all contrasts we performed statistical comparisons at four levels: (1) taxonomic profile at genus level from 16S rRNA data (biopsy samples); (2) taxonomic profile at genus level from shotgun metagenome data (faecal samples); (3) taxonomic profile at the species level from shotgun metagenome data (faecal samples); and (4) functional profile represented as metabolic pathways from shotgun metagenome data (faecal samples). Beta diversity evaluation did not reveal significant general differences between the analysed study groups, nevertheless, we continued with differential feature analyses to detect some specific markers for the taxonomic or functional profiles of these groups.

PERMANOVA analysis revealed no significant impact of the reported antibiotic use in the last two months or diarrhoea in the past week on microbiome composition in our cohort; therefore, participants with such registered events were not excluded from the cohort. In some of the contrasts, the only significant factor from PERMANOVA analysis was diabetic retinopathy (registered for 45 T1D patients), which was accordingly used in MaAsLin2 analysis as a cofactor for the specific comparisons: faecal samples of T1D patients compared to healthy controls at the (1) genus level, (2) species level and (3) functional profile; (4) faecal samples of patients with progressive DKD compared to non-progressive DKD based on the functional profile.

### T1D-specific microbial signatures, compared to healthy controls

**Figure 1.**
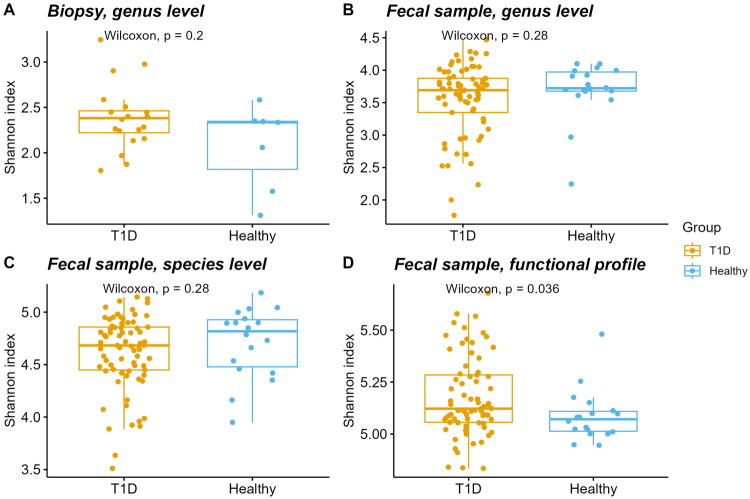
Comparison of alpha diversity based on the Shannon index between type 1 diabetes patients (T1D) and healthy individuals: (A) genus-level analysis from gut biopsy 16S rRNA library samples and (B–D) analyses from faecal metagenome library samples, including (B) genus-level, (C) species-level, and (D) functional profiles.

Firstly, we evaluated differences in Shannon index values (Figure 1), and significant differences were observed only in the diversity of functional profile when comparing T1D patients with healthy controls (Figure 1D). Interestingly, the T1D patient cohort depicted a higher dispersion of Shannon index values.

**Figure 2.**
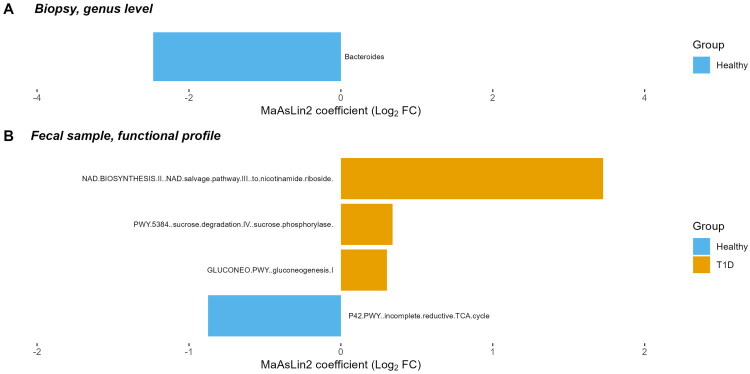
Significant microbiome feature differences when comparing type 1 diabetes (T1D) patients and healthy controls, depicted as barplots of Log2 fold change (Log2FC) coefficients from MaAsLin2 analysis (A) for bacterial genera identified in gut biopsy 16S rRNA library samples and (B) functional profiles identified in faecal metagenome library samples.

When comparing the taxonomic and functional profiles between the T1D patients and the healthy individuals, we observed that the bacterial genus *Bacteroides* was significantly enriched in the intestinal biopsy samples of the healthy control group (Figure 2A). Additionally, in the faecal metagenome library samples, NAD salvage pathway III (to nicotinamide riboside), sucrose degradation IV (sucrose phosphorylase) and gluconeogenesis I functional pathways were significantly enriched in the T1D patient group, while the incomplete reductive TCA cycle pathway was enriched in the healthy control group (Figure 2B). We did not observe any significant differences at the taxonomic level within the shotgun metagenomic data from faecal samples between the T1D patient group and healthy controls.

In addition, in faecal samples, diabetic retinopathy (DR) was identified as a significant factor in both taxonomic and functional analyses. DR was included as a cofactor in the MaAsLin2 model, which revealed additional associations related to DR. At the genus level, DR was positively associated with the genera *HGM13862*, *Bacteroides* and *CAG-56* and negatively associated with the genus *Bifidobacterium* (Supplementary Figure 1). At the species level, DR was positively associated with 8 species, such as *Enterocolitis asparagiformis*, *Mediterraneibacter torques* and *Eisenbergiella tayi* and negatively associated with 14 species, including *Bifidobacterium adolescentis*, *Bifidobacterium ruminantium* and *Bifidobacterium catenulatum* (Supplementary Figure 2). At the functional profile level, DR was positively associated with 75 pathways, including phospholipases, octane oxidation and heterolactic fermentation and negatively associated with 52 pathways, such as the bifidobacterium shunt, superpathway of L-alanine biosynthesis and superpathway of L-cysteine biosynthesis (mammalian) (Supplementary Figure 3).

### Impact of progressive DKD on gut microbiome

**Figure 3.**
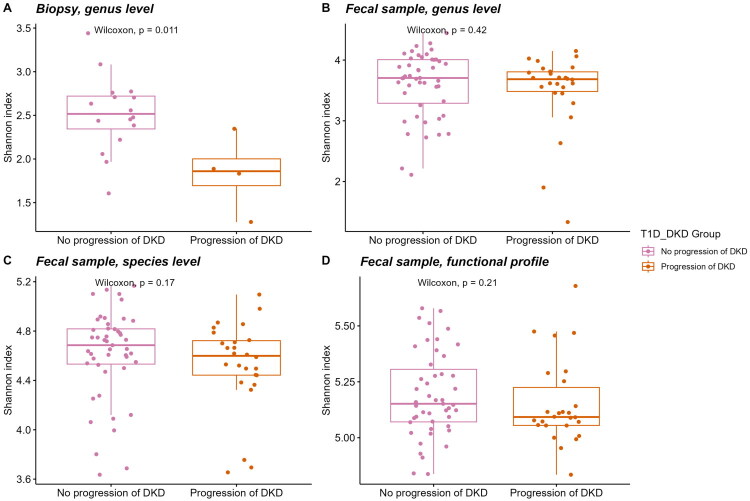
Comparison of alpha diversity based on the Shannon index between type 1 diabetes patients (T1D) with and without progression of diabetic kidney disease (DKD): (A) genus-level analysis from gut biopsy 16S rRNA library samples and (B-D) analyses from faecal metagenome library samples, including (B) genus-level, (C) species-level and (D) functional profiles.

Secondly, we focused on microbiome feature differences in T1D patients with progressive DKD and without progressive DKD. When evaluating the Shannon indices, only in the genus-level gut biopsy 16S rRNA library samples there was a statistically significant difference (Figure 3A). The progressive DKD was generally characterized by lower alpha diversity in all contrasts (Figure 3A–D).

**Figure 4.**
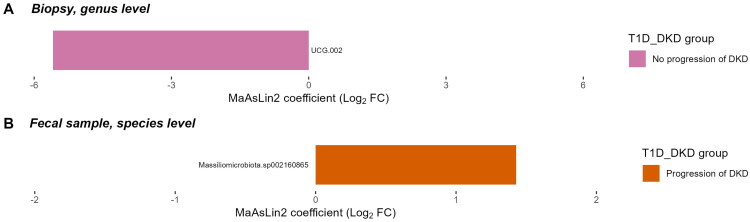
Significant microbiome feature differences when comparing type 1 diabetes (T1D) patients with progressive and non-progressive diabetic kidney disease (DKD), depicted as barplots of Log2 fold change (Log2FC) coefficients from MaAsLin2 analysis (A) for bacterial genera identified in gut biopsy 16S rRNA library samples and (B) microbial species identified in faecal metagenome library samples.

**Figure 5.**
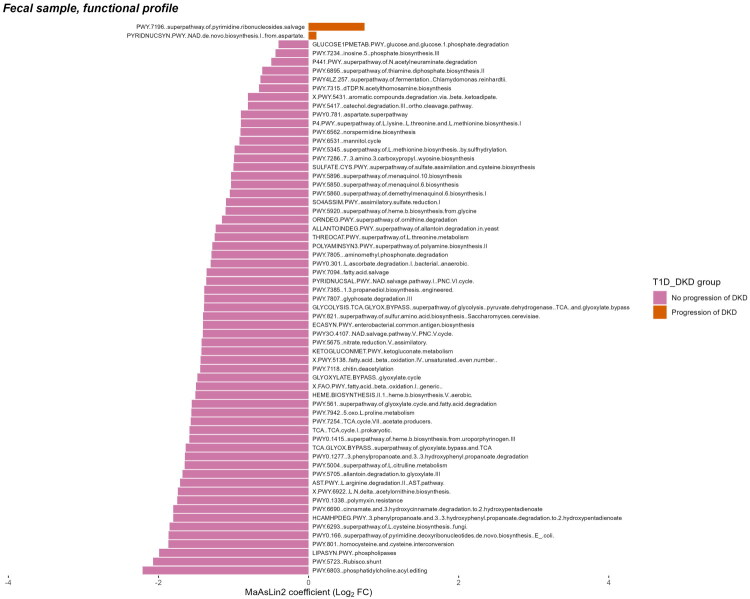
Significant microbiome feature differences when comparing type 1 diabetes (T1D) patients with progressive and non-progressive diabetic kidney disease (DKD), depicted as barplots of Log2 fold change (Log2FC) coefficients from MaAsLin2 analysis for functional profiles identified in faecal metagenome library samples.

When comparing the taxonomic and functional profiles between progressive and non-progressive DKD patients, we observed that the bacterial genus *Oscillospiraceae UCG-002*, identified in the intestinal biopsy 16S rRNA library samples, was significantly enriched in the non-progressive DKD patient group (Figure 4A), whereas in the faecal metagenome library samples, the bacterial species *Massiliomicrobiota sp002160865* was significantly enriched in the progressive DKD patient group (Figure 4B). There were no significant differences at the genus level of the metagenomic data from faecal samples between progressive and non-progressive DKD patients. Additionally, in the faecal metagenome library samples, superpathway of pyrimidine ribonucleosides salvage and NAD de novo biosynthesis I (from aspartate) functional pathways were significantly enriched in the progressive DKD patient group, while phosphatidylcholine acyl editing, Rubisco shunt, phospholipases, homocysteine and cysteine interconversion, superpathway of pyrimidine deoxyribonucleotides de novo biosynthesis (*E.coli*) and 56 other pathways was enriched in the non-progressive DKD patient group (Figure 5).

Additionally, in faecal samples, DR was identified as a significant factor in functional analyses. DR was also included as a cofactor in the MaAsLin2 model, which revealed additional associations related to DR. DR was positively associated with phospholipases, phosphatidylcholine acyl editing, heterolactic fermentation and 121 other pathways and negatively associated with bifidobacterium shunt, superpathway of pyrimidine ribonucleosides salvage, superpathway of L-alanine biosynthesis and 61 other pathways (Supplementary Figure 4).

### Blood glucose level and its regulation-related differences

**Figure 6.**
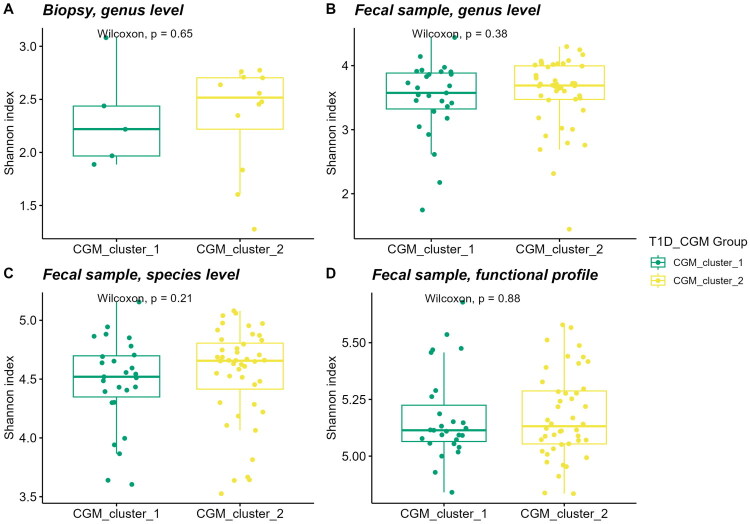
Comparison of alpha diversity based on the Shannon index between type 1 diabetes patients (T1D) in two cluster groups based on continuous glucose monitoring (CGM) data: (A) genus-level analysis from gut biopsy 16S rRNA library samples and (B–D) analyses from faecal metagenome library samples, including (B) genus-level, (C) species-level and (D) functional profiles.

Patients in the CGM cluster 1 subgroup exhibited higher average glucose levels (12,63 ± 2,55 mmol/L), lower glucose variability (CV = 36,56 ± 5,15%) and a greater percentage of time above target range (%TAR = 67,70 ± 14,66%), whereas those in CGM cluster 2 had lower average glucose levels (8,71 ± 1,63 mmol/L), higher glucose variability (CV = 42,97 ± 7,38%) and spent less time above target range (%TAR = 31,36 ± 14,41%). When comparing alpha diversity, CGM cluster 2 demonstrated higher Shannon index values; however, the differences in all contrasts did not reach statistical significance (Figure 6).

Similarly, when comparing the taxonomic and functional profiles between T1D patients in CGM cluster 1 and CGM cluster 2, there were no statistically significant differences in MaAsLin analysis after correction. However, such species as *Butyricimonas faecalis*, CAG-488 sp000434915 (*Acutalibacteraceae* family) and *Sutterella wadsworthensis* were observed to be associated with CGM cluster 2 and CAG-873 sp001701165 (*Muribaculaceae* family) with CGM cluster 1 before performing multiple correction (*p* < .05, *q* > 0.25). In total, 12 species were found to be associated with CGM cluster 1 and 44 with CGM cluster 2 before multiple correction.

### Correlation between paired gut biopsy 16S rRNA and faecal metagenome samples

**Figure 7.**
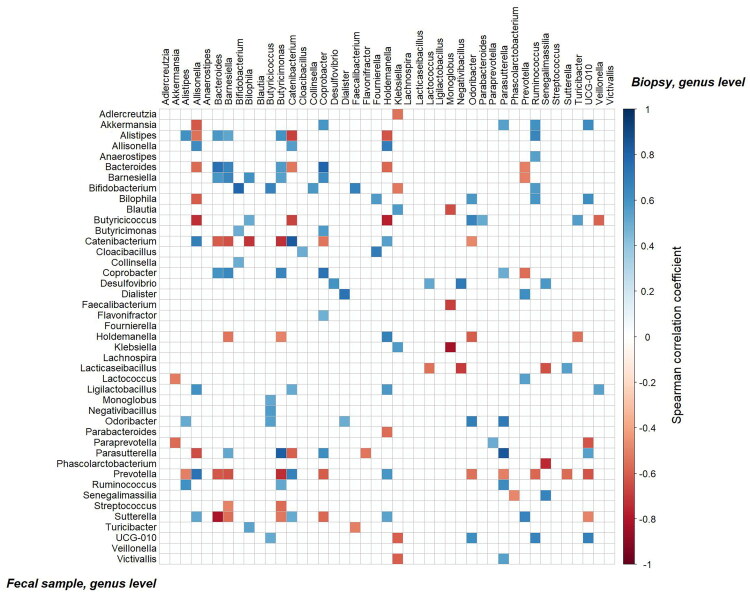
Correlation matrix (spearman) between the shared 43 genera that were present both in biopsy and faecal samples of T1D patients. Positive correlations are displayed in graded blue colours and negative correlations in graded red colours, only statistically significant correlations are depicted.

Finally, we decided to analyse the possible correlations between data from biopsy samples and faecal samples within the T1D cohort (*N* = 17) by focusing on taxa (43 genera) that were observed in both sample types at the genus level (Figure 7). In total, 94 positive and 61 negative statistically significant correlations (*p* < .05) were identified. The strongest positive correlations were found for the *Parasutterella* genus, which showed a significant correlation between biopsy and faecal samples (ρ = 0.841, *p* = 2.32 × 10^−5^), followed by *Catenibacterium*, which also exhibited a strong correlation between biopsy and faecal samples (ρ = 0.832, *p* = 3.49 × 10^−5^). Additionally, *Butyricimonas* in biopsy samples was strongly correlated with *Parasutterella* in faecal samples (ρ = 0.815, *p* = 6.73 × 10^−5^). The strongest negative correlations were observed between *Monoglobus* in biopsy samples and *Klebsiella* in faecal samples (ρ = −0.830, *p* = 3.73 × 10^−5^), *Bacteroides* in biopsy samples and *Sutterella* in faecal samples (ρ = −0.814, *p* = 9.09 × 10^−5^) and *Holdemanella* in biopsy samples and *Butyricicoccus* in faecal samples (ρ = −0.779, *p* = 2.26 × 10^−4^).

Additionally, we also performed Spearman correlation analysis on all genera found in biopsy and faecal samples, including 126 genera from biopsy samples and 379 genera from faecal samples of T1D patients (*N* = 17) (Supplementary Figure 5). In total, 2737 positive and 929 negative statistically significant correlations (*p* < .05) were identified. Among the strongest positive correlations, *Lachnospiraceae [Eubacterium] hallii group* in biopsy samples exhibited the highest correlation with *SFJ001* genus in faecal samples (ρ = 0.902, *p* = 0), followed by *UCG-002* in biopsy samples with *PeH17* in faecal samples (ρ = 0.861, *p* = 9.24 × 10^−6^) and *UCG-010* in biopsy samples with *UMGS1603* in faecal samples (ρ = 0.859, *p* = 1.03 × 10^−5^). Conversely, the strongest negative correlations were observed between *Butyricimonas* in biopsy samples and *Absicoccus* in faecal samples (ρ = −0.845, *p* = 1.99 × 10^−5^), *Monoglobus* in biopsy samples and *Klebsiella* in faecal samples (ρ = −0.830, *p* = 3.73 × 10^−5^) and *Bacteroides* in biopsy samples and *Sutterella* in faecal samples (ρ = −0.814, *p* = 9.09 × 10^−5^).

## Discussion

Our study employed both 16S rRNA amplicon and shotgun metagenomic sequencing to analyse the gut microbiome in intestinal biopsy (sigmoid colon) and faecal samples respectively, revealing distinct microbial taxonomic and functional patterns in T1D patients. These findings provide significant insights into the relationship between the gut microbiome, T1D progression, diabetic kidney disease (DKD) and even diabetic retinopathy (DR), offering valuable context for the ongoing exploration of microbial contributions to diabetes and its complications. Moreover, this is the first study combining these both sample types in the context of T1D.

We found no significant differences in the alpha diversity (expressed as Shannon index) at the genus or species levels for biopsy and faecal samples when comparing T1D patients with healthy controls (Figure 1A–C), aligning with previous studies [32]. However, the T1D group exhibited greater dispersion in alpha diversity metrics compared to the healthy controls, suggesting increased variability within this group. Functional profiles from faecal metagenomes showed a significantly higher alpha diversity in T1D patients (Figure 1D, *p* = .036) supporting the idea that disease-associated microbiomes adapt functionally despite taxonomic stability [33].

When comparing DKD progressors and non-progressors biopsy samples from DKD progressors showed a significant reduction in alpha diversity (Figure 3A, *p* = .011), indicating a loss of microbial richness linked to disease progression. However, no significant differences were observed in faecal samples, which may reflect the distinct roles of microbiota at the mucosal surface versus the lumen. This also highlights the limitations of faecal samples in representing mucosal-associated microbiota, as faecal and mucosal communities may differ in composition and function. While Nowicki et al. [34] reported significant compositional differences between faecal and mucosal biopsy samples, emphasizing that faecal samples may not fully capture the localized microbial dynamics at the mucosal surface, Mukhopadhya et al. [35] found no substantial differences in bacterial richness and diversity. This discrepancy underscores the influence of sample type, disease context and methodology, emphasizing the need for careful interpretation of microbiome data, particularly when using faecal samples as a substitute for mucosal microbiota.

MaAsLin2 analysis identified significant microbial taxa and functional differences between T1D patients and healthy controls. In biopsy samples, a reduced relative abundance of the genus *Bacteroides* was observed in T1D patients compared to healthy controls (Figure 2A). *Bacteroides* is known for its role in maintaining gut barrier integrity and modulating immune responses [36]. Although studies on biopsy samples are limited, findings regarding *Bacteroides* in T1D microbiome studies are mixed. Our findings suggest specific disruptions in the mucosal microbiome, potentially indicating that T1D involves localized gut dysbiosis that is distinct from luminal microbiota alterations. Further research comparing mucosal and faecal microbiota is needed to elucidate these differences [37,38]. In faecal samples, functional pathways, including NAD salvage pathways and gluconeogenesis, were significantly enriched in T1D patients (Figure 2B). These pathways are crucial for cellular energy metabolism and stress responses. NAD salvage pathways are particularly important for maintaining cellular NAD+ levels, which influence energy homeostasis and immune regulation, while gluconeogenesis plays a role in glucose production during metabolic stress. These findings align with prior research on NAD metabolism and gluconeogenesis in diabetes, emphasizing their roles in systemic metabolic alterations [39]. Such metabolic disruptions may reflect systemic changes associated with T1D and highlight potential therapeutic targets.

In biopsy samples, the genus *Oscillospiraceae UCG-002* was more abundant in the non-progressive DKD group (Figure 4A), suggesting a potential role in modulating kidney disease progression. In previous findings, the association of this genus with DKD has not been described; however, it has been investigated that the genus *Oscillospiraceae UCG-002* is negatively associated with the insulin resistance index (HOMA-IR) and is linked to mechanisms involving sulphate-reducing bacteria, increased intestinal gluconeogenesis and acetate production [40]. These results align with our findings and suggest that *Oscillospiraceae UCG-002* may influence metabolic and inflammatory pathways critical to kidney health. Further investigations are necessary to determine the specific mechanisms linking this genus to non-progressive DKD and its potential role in disease modulation. Conversely, in faecal samples, enrichment of *Massiliomicrobiota sp002160865* was observed in the progressive DKD group (Figure 4B). This novel finding could reflect adaptive shifts in microbial composition due to systemic inflammation or altered intestinal permeability associated with DKD progression, as the family of this genus - *Erysipelotrichaceae -* has been associated with these processes [41]. While *Massiliomicrobiota* has been sparsely studied in the context of faecal microbiomes, this observation underscores the need for further research into its functional role in DKD.

In faecal samples, the functional analysis highlighted significant enrichment of NAD de novo biosynthesis (from aspartate) and pyrimidine ribonucleoside salvage pathways in progressive DKD patients (Figure 5). The NAD biosynthesis pathway is crucial for energy metabolism and cellular repair mechanisms, while pyrimidine salvage supports nucleotide turnover and DNA repair. These findings are consistent with studies indicating that reduced NAD+ levels in DKD impair mitochondrial function and increase oxidative stress and inflammation, exacerbating disease progression [42]. Similarly, disruptions in pyrimidine salvage pathways have been associated with kidney pathology due to the accumulation of toxic metabolites and compromised DNA repair [43]. These metabolic disturbances highlight potential therapeutic targets aimed at restoring NAD+ levels or enhancing nucleotide salvage to mitigate renal injury and inflammation. Interestingly, some of the metabolic pathways enriched in the group of T1D patients without DKD progression are often related to enhanced renal function or mechanisms involved in protection against diabetic nephropathy. For example, L-cysteine is a precursor for glutathione, which is an important antioxidant and has demonstrated protective effects from diabetic nephropathy and neuropathy in rats [44]. Pathways such as PWY-5431 (aromatic compound degradation *via* beta-ketoadipate pathway) and PWY-5417 (catechol degradation) are involved in the degradation of aromatic amino acid and polyphenols, producing microbiome-derived metabolites that are beneficial for host health [45]. Others are related to metabolites with diverse or unclear functions and effects on kidney health, such as 3-phenylpropanoate and 3-(3-hydroxyphenyl)propanoate degradation to 2-hydroxypentadienoate which among other products yields succinate, a significant metabolite in diabetes pathological mechanisms and DKD development [46]. Similarly, the cinnamate and 3-hydroxycinnamate degradation to 2-hydroxypentadienoate yields also fumarate, which has also been described as a key mediator of DKD pathogenesis [47]. Thus these results support the possible role of gut microbiome and its interaction with host on the development of diabetes complications.

Cluster analyses based on continuous glucose monitoring (CGM) metrics revealed no significant differences in alpha diversity or other microbial features between the two clusters indicating similar microbiome profiles across glycaemic groups (Figure 6A–D). This may indicate that microbial changes in T1D are more closely tied to other factors, such as disease progression or complications, rather than glycaemic variability alone. Further studies are needed to investigate the potential influences of glycaemic profiles on the microbiome using larger cohorts and integrative methods.

Interestingly, the diagnosis of DR was a significant factor associated with differential both taxonomic and functional microbiome composition. As this was not the focus of this study, DR was used as a cofactor of statistical models. Nevertheless, we obtained data about microbial features significantly associated with DR, which were highly consistent with available data, such as increased abundance of *Bacteroidetes* and decreased abundance of *Bifidobacterium* in patients with DR [48].

The analysis of correlations between genera in faecal and biopsy samples revealed insights into the relationship between mucosal and faecal microbiota. However, since biopsy samples were collected more than three months after faecal samples, and assuming no major lifestyle and health changes occurred, results should be interpreted with caution. A total of 43 shared genera were identified between both sample types, with correlations assessed to evaluate microbial overlap and potential clinical implications (Figure 7).

Among the strongest positive correlations, *Parasutterella* and *Catenibacterium* showed a consistent relationship between faecal and biopsy samples, suggesting that their abundance in faecal samples reliably reflects their presence in biopsy samples. *Parasutterella* is involved in glucose regulation in type 2 diabetes (T2D) by consuming L-cysteine in rodents and has been linked to weight loss-associated microbial shifts in humans with T2D [49], while *Catenibacterium* contributes to short-chain fatty acid (SCFA) production, particularly butyrate, which supports gut health and systemic inflammation regulation [50].

In contrast, negative correlations highlighted potentially distinct ecological and functional differences between taxa in the two compartments, with the strongest inverse relationship observed between *Monoglobus* and *Klebsiella*. This may reflect their contrasting roles, as *Klebsiella* is associated with opportunistic infections and inflammation, while *Monoglobus* contributes to fibre fermentation. Elevated *Klebsiella* abundance has been linked to intestinal inflammation and disruption of gut homeostasis, highlighting its pro-inflammatory potential [51]. Additionally, *Klebsiella* genes, particularly *K. oxytoca* in circulation, have been proposed as biomarkers for renal function decline in DKD patients [52]. Conversely, *Monoglobus* plays a protective role by fermenting dietary fibres and producing beneficial metabolites such as butyrate, which supports gut barrier integrity and reduces inflammation [53].

The dual sequencing approach combining 16S rRNA and shotgun metagenomics has proven essential in capturing a comprehensive view of microbial and metabolic changes associated with T1D. Furthermore, the inclusion of both faecal and biopsy samples highlights the need to account for localized mucosal dynamics alongside luminal microbial profiles, as these sample types exhibit complementary yet distinct features. Despite the observed taxonomic stability, the functional diversity shifts observed in T1D and DKD highlight the adaptability of microbial communities in response to disease-associated stressors. This functional plasticity suggests that interventions targeting microbial metabolic pathways, rather than specific taxa, may be more effective in mitigating disease progression.

Our study also has some limitations, such as the number of controls could be higher, however, those used in this analysis have been matched by age, gender and BMI to the T1D cohort. This matching was confirmed by statistical comparisons showing no significant differences between the groups. Although fewer healthy controls were included due to limited availability of biopsy samples in this population, the relative homogeneity of the control group further reduces the potential for confounding. Together, these factors support the validity of subsequent microbiome analyses. Unfortunately, only a part of the study participants had both sample types (biopsy and faecal) available, as biopsy sample collection is invasive and should be justified by medical reasons. While our metagenomic analyses suggest potential functional alterations in the gut microbiome, including pathways such as NAD synthesis, the lack of complementary metabolomic data limits our ability to confirm their impact on host metabolism or DKD progression. Future research should focus on validating these findings in larger and more diverse cohorts to better account for demographic and environmental influences on the gut microbiome. In-depth studies are essential to establish causative relationships between microbiota alterations and disease progression. Targeted investigations are also needed to elucidate the specific roles of key taxa and functional pathways in disease aetiology and progression. Furthermore, the integration of multi-omics data, including host genetics, metabolomics and proteomics, with microbiome analyses will enhance the understanding of the complex interactions driving T1D and its complications, such as DKD and DR.

## Conclusions

In conclusion, this study presents that the gut microbiome exhibits distinct functional disruptions in T1D patients, with implications for disease progression and specifically DKD. While taxonomic diversity remains largely similar, the observed functional shifts underscore the importance of considering metabolic pathways in understanding disease-associated dysbiosis. Similarly, the novel data could highlight some population-specific signatures. The findings also emphasise the value of combining biopsy and faecal sample analyses to capture comprehensive microbial dynamics, particularly in relation to mucosal-specific alterations. By linking microbiome composition and functionality to clinical outcomes, this research provides a foundation for the future development of microbiome-based diagnostics and therapeutics. Addressing the identified microbial and functional alterations offers promising avenues for improving the management of T1D and preventing its complications, ultimately contributing to better patient outcomes.

## Supplementary Material

Supplementary Figure 5.jpg

Supplementary Figure 1.jpg

Supplementary_Table1 and Table 2_primer_index_sequences.docx

Supplementary Figure 3.jpg

Supplementary figure legends.docx

Supplementary Figure 4.jpg

Supplementary Figure 2.jpg

## Data Availability

All V3–V4 16S rRNA gene amplicon and shotgun metagenomic sequences are available from the European Nucleotide Archive (ENA) under the accession number PRJEB85321. Additional data will be available upon reasonable request.
