# Supplementary figures and images for: Dual-approach analysis of gut microbiome in patients with type 1 diabetes and diabetic kidney disease
Source: Ann Med. 2025 Jul 26;57(1):2531254. doi: 10.1080/07853890.2025.2531254 (PMC12302470; doi:10.1080/07853890.2025.2531254)

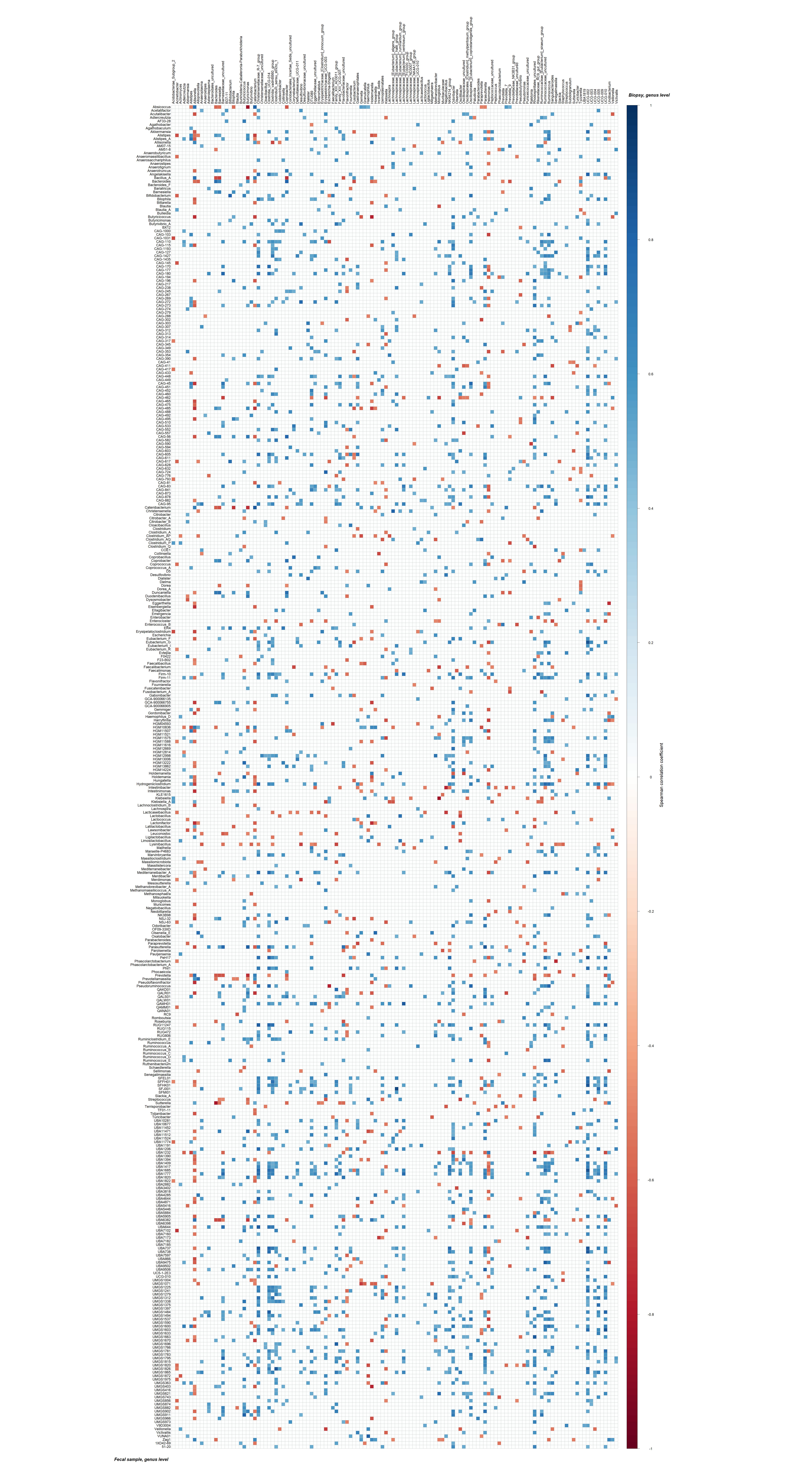

Supplement: Supplementary Figure 5.jpg [file IANN_A_2531254_SM7760.jpg]

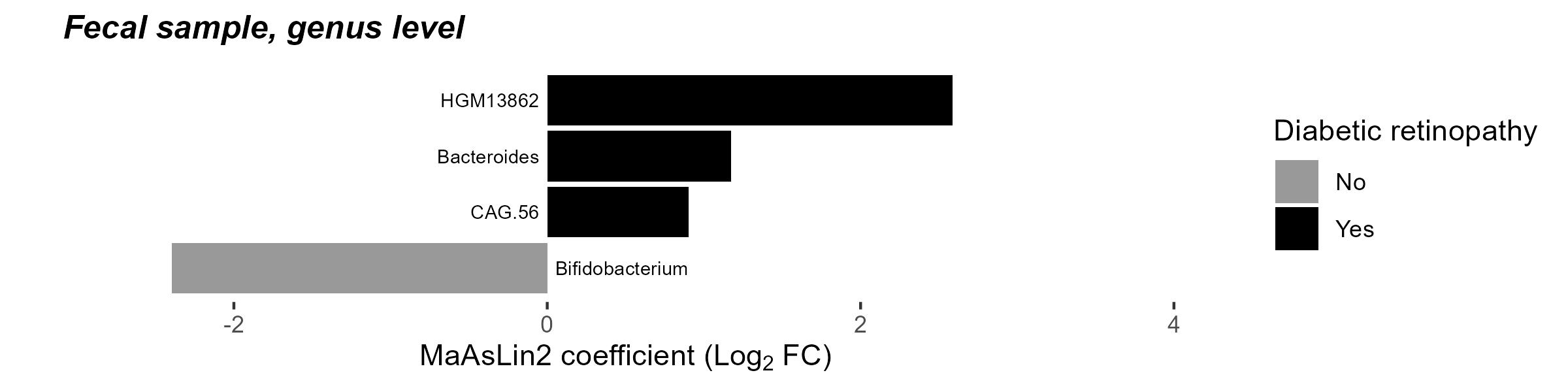

Supplement: Supplementary Figure 1.jpg [file IANN_A_2531254_SM7759.jpg]

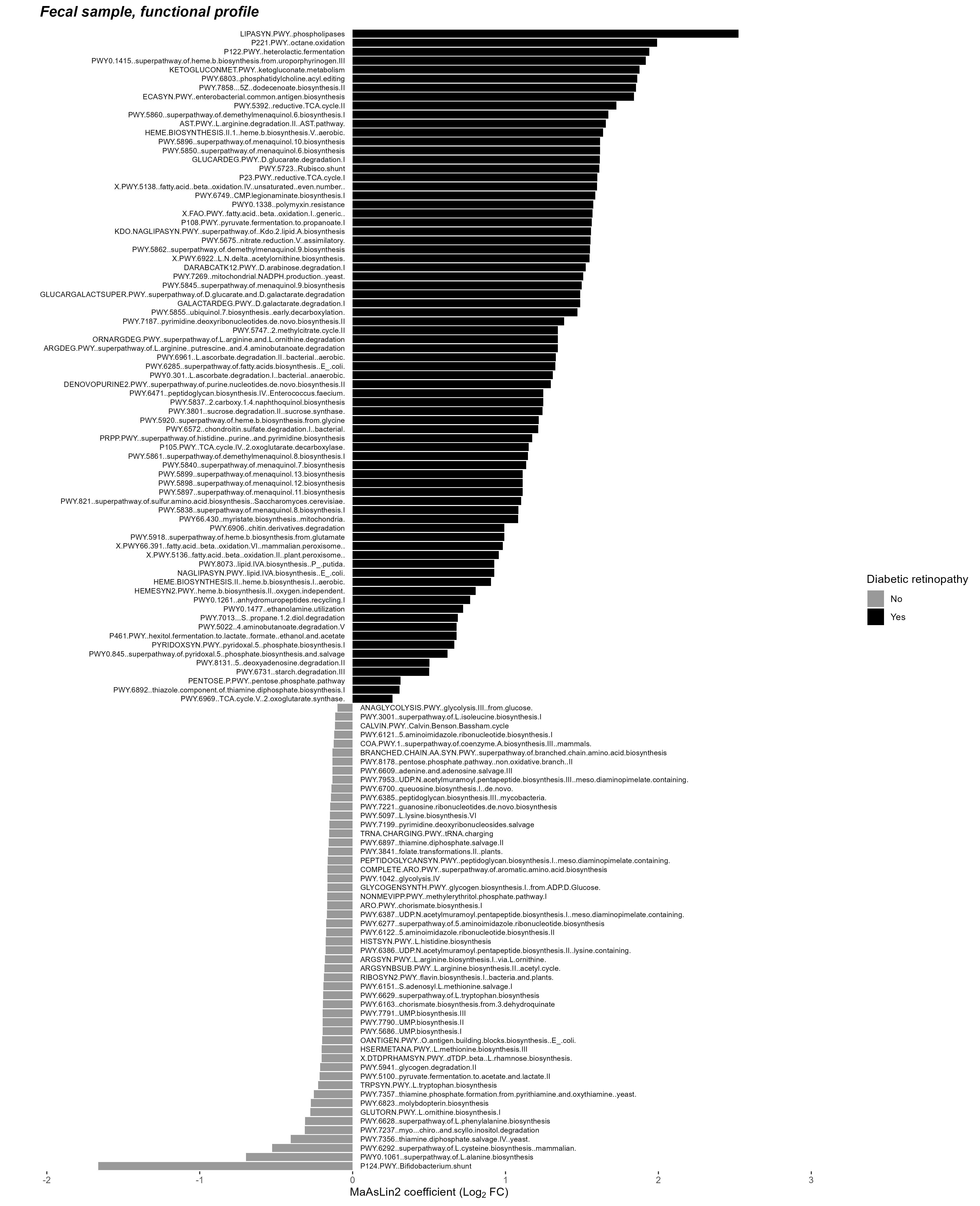

Supplement: Supplementary Figure 3.jpg [file IANN_A_2531254_SM7757.jpg]

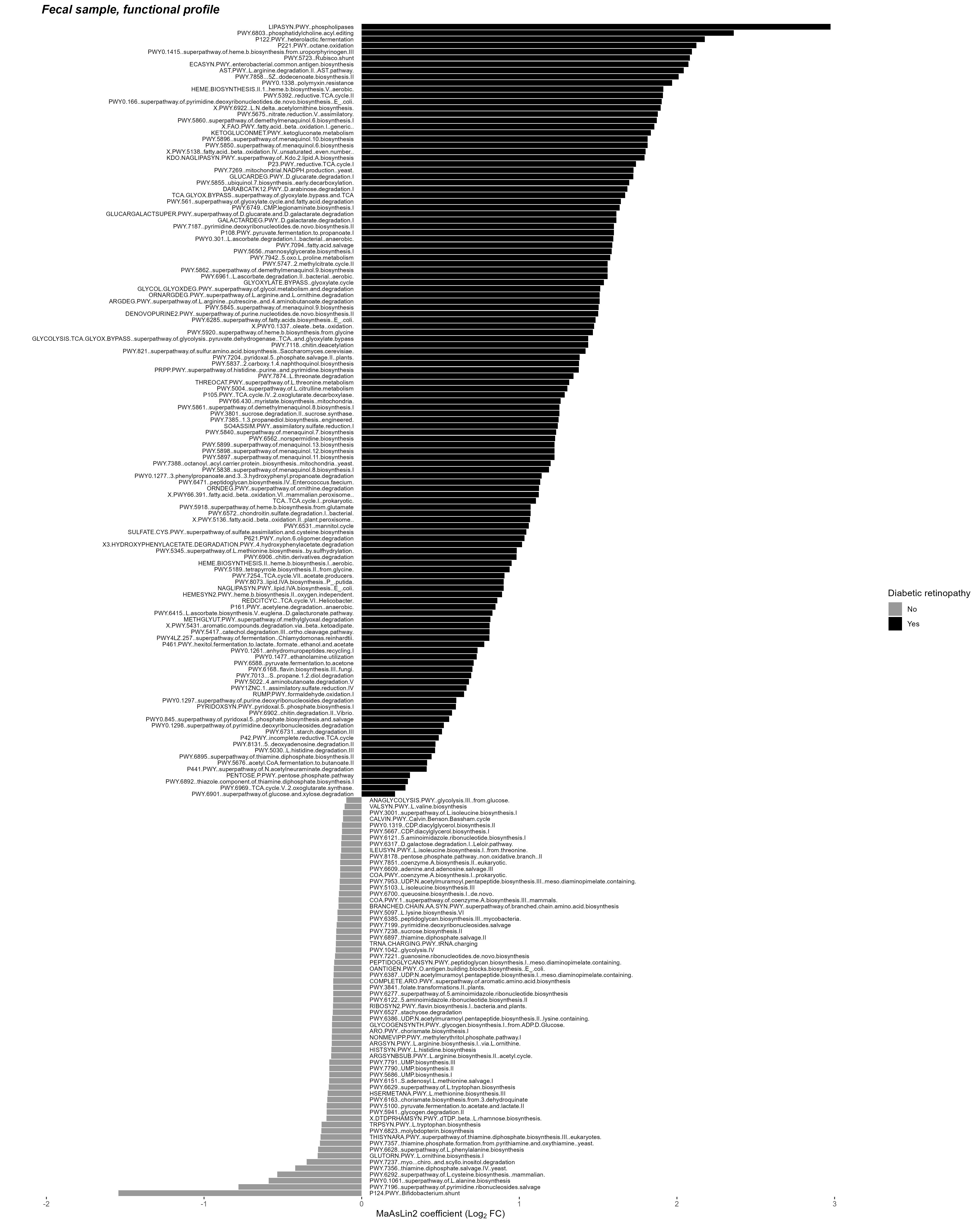

Supplement: Supplementary Figure 4.jpg [file IANN_A_2531254_SM7755.jpg]

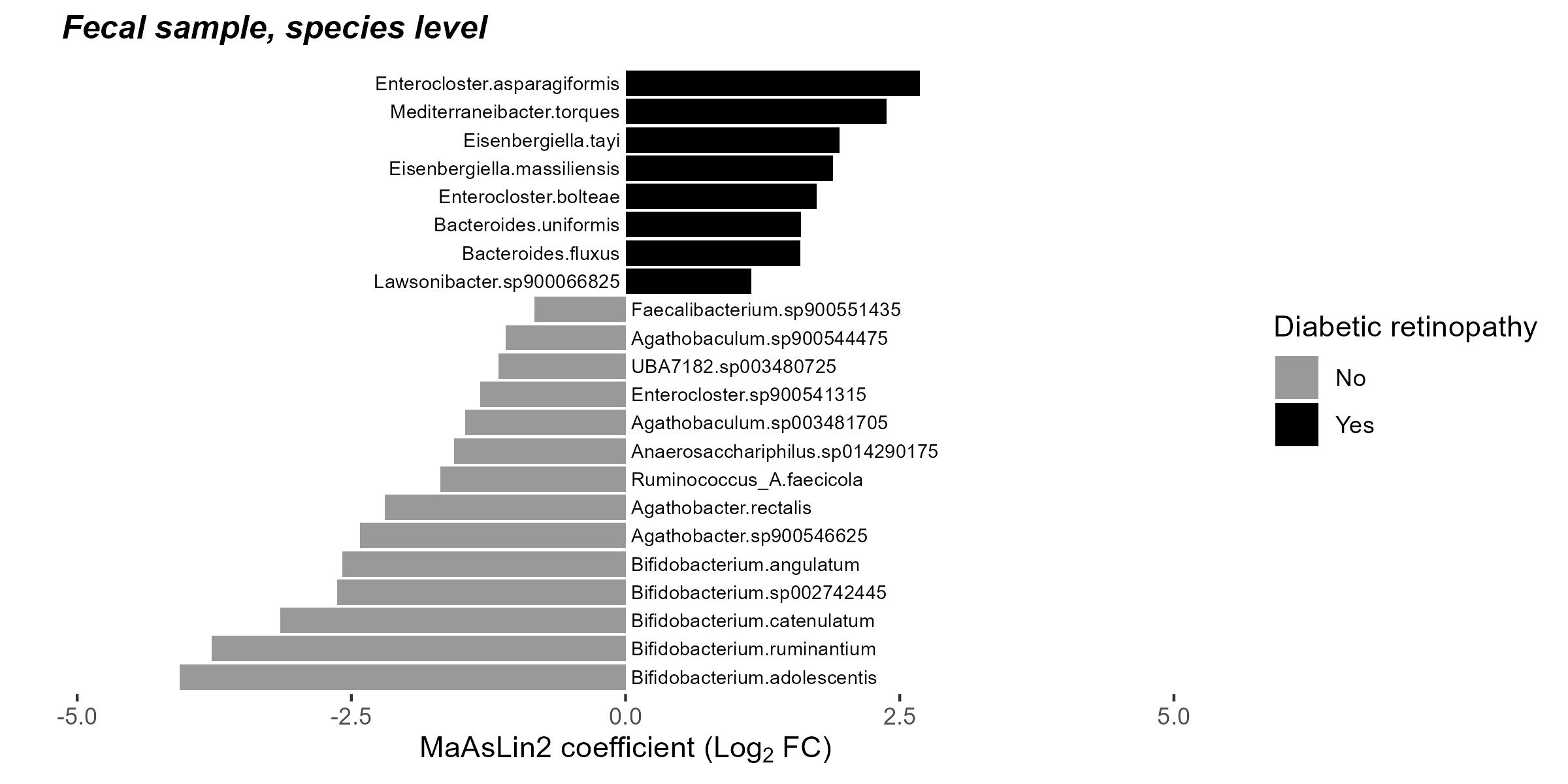

Supplement: Supplementary Figure 2.jpg [file IANN_A_2531254_SM7754.jpg]
