# Supplementary material for: Dual-approach analysis of gut microbiome in patients with type 1 diabetes and diabetic kidney disease
Source: Ann Med. 2025 Jul 26;57(1):2531254. doi: 10.1080/07853890.2025.2531254 (PMC12302470; doi:10.1080/07853890.2025.2531254)
Supplement: Supplementary_Table1 and Table 2_primer_index_sequences.docx [file IANN_A_2531254_SM7758.docx]

**Supplementary table 1.**

Used primer sequences

| **Primer** | **Sequence** |
| --- | --- |
| ci5_16S_V3_Fw341F | TCGTCGGCAGCGTCAGATGTGTATAAGAGACAGNNNNNNCCTACGGGNGGCWGCAG |
| ci7_16S_V4_Rs805R | GTCTCGTGGGCTCGGAGATGTGTATAAGAGACAGNNNNNNGACTACHVGGGTATCTAATCC |

Abbreviations: A – adenine, T – thymine, G – guanine, C – cytosine, N – any base, W – adenine or thymine, H – adenine, cytosine or thymine, V – adenine, cytosine or guanine.

**Supplementary table 2.**

Used index primer sequences

| **Index primer** | **Sequence** |
| --- | --- |
| i7_Adapter_N701 | CAAGCAGAAGACGGCATACGAGATTAAGGCGAGTCTCGTGGGCTCGGAGATGTGTATAAGAGACAG |
| i7_Adapter_N702 | CAAGCAGAAGACGGCATACGAGATCGTACTAGGTCTCGTGGGCTCGGAGATGTGTATAAGAGACAG |
| i7_Adapter_N703 | CAAGCAGAAGACGGCATACGAGATAGGCAGAAGTCTCGTGGGCTCGGAGATGTGTATAAGAGACAG |
| i7_Adapter_N704 | CAAGCAGAAGACGGCATACGAGATTCCTGAGCGTCTCGTGGGCTCGGAGATGTGTATAAGAGACAG |
| i7_Adapter_N705 | CAAGCAGAAGACGGCATACGAGATGGACTCCTGTCTCGTGGGCTCGGAGATGTGTATAAGAGACAG |
| i7_Adapter_N706 | CAAGCAGAAGACGGCATACGAGATTAGGCATGGTCTCGTGGGCTCGGAGATGTGTATAAGAGACAG |
| i7_Adapter_N707 | CAAGCAGAAGACGGCATACGAGATCTCTCTACGTCTCGTGGGCTCGGAGATGTGTATAAGAGACAG |
| i7_Adapter_N708 | CAAGCAGAAGACGGCATACGAGATCAGAGAGGGTCTCGTGGGCTCGGAGATGTGTATAAGAGACAG |
| i7_Adapter_N709 | CAAGCAGAAGACGGCATACGAGATGCTACGCTGTCTCGTGGGCTCGGAGATGTGTATAAGAGACAG |
| i7_Adapter_N710 | CAAGCAGAAGACGGCATACGAGATCGAGGCTGGTCTCGTGGGCTCGGAGATGTGTATAAGAGACAG |
| i7_Adapter_N711 | CAAGCAGAAGACGGCATACGAGATAAGAGGCAGTCTCGTGGGCTCGGAGATGTGTATAAGAGACAG |
| i7_Adapter_N712 | CAAGCAGAAGACGGCATACGAGATGTAGAGGAGTCTCGTGGGCTCGGAGATGTGTATAAGAGACAG |
| i5_Adapter_E501 | AATGATACGGCGACCACCGAGATCTACACTAGATCGCTCGTCGGCAGCGTCAGATGTGTATAAGAGACAG |
| i5_Adapter_E502 | AATGATACGGCGACCACCGAGATCTACACCTCTCTATTCGTCGGCAGCGTCAGATGTGTATAAGAGACAG |
| i5_Adapter_E503 | AATGATACGGCGACCACCGAGATCTACACTATCCTCTTCGTCGGCAGCGTCAGATGTGTATAAGAGACAG |
| i5_Adapter_E504 | AATGATACGGCGACCACCGAGATCTACACAGAGTAGATCGTCGGCAGCGTCAGATGTGTATAAGAGACAG |
| i5_Adapter_E505 | AATGATACGGCGACCACCGAGATCTACACGTAAGGAGTCGTCGGCAGCGTCAGATGTGTATAAGAGACAG |
| i5_Adapter_E506 | AATGATACGGCGACCACCGAGATCTACACACTGCATATCGTCGGCAGCGTCAGATGTGTATAAGAGACAG |
| i5_Adapter_E507 | AATGATACGGCGACCACCGAGATCTACACAAGGAGTATCGTCGGCAGCGTCAGATGTGTATAAGAGACAG |
| i5_Adapter_E508 | AATGATACGGCGACCACCGAGATCTACACCTAAGCCTTCGTCGGCAGCGTCAGATGTGTATAAGAGACAG |

Abbreviations: A – adenine, T – thymine, G – guanine, C – cytosine.
