# Supplementary material for: Dual-approach analysis of gut microbiome in patients with type 1 diabetes and diabetic kidney disease
Source: Ann Med. 2025 Jul 26;57(1):2531254. doi: 10.1080/07853890.2025.2531254 (PMC12302470; doi:10.1080/07853890.2025.2531254)
Supplement: Supplementary figure legends.docx [file IANN_A_2531254_SM7756.docx]

**Supplementary figure 1.** Significant microbiome feature differences associated with diabetic retinopathy (DR) when comparing type 1 diabetes (T1D) patients and healthy controls with DR included as a cofactor in MaAsLin2 analysis. Results are depicted as barplots of Log2 fold change (Log2FC) coefficients for bacterial genera identified in fecal metagenome library samples.

**Supplementary figure 2.** Significant microbiome feature differences associated with diabetic retinopathy (DR) when comparing type 1 diabetes (T1D) patients and healthy controls with DR included as a cofactor in MaAsLin2 analysis. Results are depicted as barplots of Log2 fold change (Log2FC) coefficients for bacterial species identified in fecal metagenome library samples.

**Supplementary figure 3.** Significant microbiome feature differences associated with diabetic retinopathy (DR) when comparing type 1 diabetes (T1D) patients and healthy controls with DR included as a cofactor in MaAsLin2 analysis. Results are depicted as barplots of Log2 fold change (Log2FC) coefficients for functional profiles identified in fecal metagenome library samples.

**Supplementary figure 4.** Significant microbiome feature differences associated with diabetic retinopathy (DR) when comparing type 1 diabetes (T1D) patients with progressive and non-progressive diabetic kidney disease (DKD) with DR included as a cofactor in MaAsLin2 analysis. Results are depicted as barplots of Log2 fold change (Log2FC) coefficients for functional profiles identified in fecal metagenome library samples.

**Supplementary figure 5.** Spearman correlation matrix between all genera identified in biopsy and fecal samples of T1D patients. Positive correlations are shown in graded blue colors, while negative correlations are displayed in graded red colors.
